# Blunted ventral striatal responses to anticipated rewards foreshadow problematic drug use in novelty-seeking adolescents

**DOI:** 10.1038/ncomms14140

**Published:** 2017-02-21

**Authors:** Christian Büchel, Jan Peters, Tobias Banaschewski, Arun L. W. Bokde, Uli Bromberg, Patricia J. Conrod, Herta Flor, Dimitri Papadopoulos, Hugh Garavan, Penny Gowland, Andreas Heinz, Henrik Walter, Bernd Ittermann, Karl Mann, Jean-Luc Martinot, Marie-Laure Paillère-Martinot, Frauke Nees, Tomas Paus, Zdenka Pausova, Luise Poustka, Marcella Rietschel, Trevor W. Robbins, Michael N. Smolka, Juergen Gallinat, Gunter Schumann, Brian Knutson, the IMAGEN consortium, Mercedes Arroyo, Eric Artiges, Semiha Aydin, Christine Bach, Alexis Barbot, Gareth Barker, Ruediger Bruehl, Anna Cattrell, Patrick Constant, Hans Crombag, Katharina Czech, Jeffrey Dalley, Benjamin Decideur, Sylvane Desrivieres, Tahmine Fadai, Mira Fauth-Buhler, Jianfeng Feng, Irinia Filippi, Vincent Frouin, Birgit Fuchs, Isabel Gemmeke, Alexander Genauck, Eanna Hanratty, Bert Heinrichs, Nadja Heym, Thomas Hubner, Albrecht Ihlenfeld, Alex Ing, James Ireland, Tianye Jia, Jennifer Jones, Sarah Jurk, Mehri Kaviani, Arno Klaassen, Johann Kruschwitz, Christophe Lalanne, Dirk Lanzerath, Mark Lathrop, Claire Lawrence, Hervé Lemaitre, Christine Macare, Catherine Mallik, Adam Mar, Lourdes Martinez-Medina, Eva Mennigen, Fabiana Mesquita de Carvahlo, Xavier Mignon, Sabina Millenet, Ruben Miranda, Kathrin Müller, Charlotte Nymberg, Caroline Parchetka, Yolanda Pena-Oliver, Jani Pentilla, Jean-Baptiste Poline, Erin Burke Quinlan, Michael Rapp, Stephan Ripke, Tamzin Ripley, Gabriel Robert, John Rogers, Alexander Romanowski, Barbara Ruggeri, Christine Schmäl, Dirk Schmidt, Sophia Schneider, Florian Schubert, Yannick Schwartz, Wolfgang Sommer, Rainer Spanagel, Claudia Speiser, Tade Spranger, Alicia Stedman, Dai Stephens, Nicole Strache, Andreas Ströhle, Maren Struve, Naresh Subramaniam, David Theobald, Nora Vetter, Helene Vulser, Katharina Weiss, Robert Whelan, Steve Williams, Bing Xu, Juliana Yacubian, Tao Yu, Veronika Ziesch

**Affiliations:** 1 Department of Systems Neuroscience, Universitätsklinikum Hamburg Eppendorf, 20246 Hamburg, Germany; 2 Department of Psychology, Stanford University, Stanford, California 94305, USA; 3 Department of Child and Adolescent Psychiatry and Psychiatry, Central Institute of Mental Health, Medical Faculty Mannheim, Heidelberg University, 68159 Mannheim, Germany; 4 Institute of Neuroscience and Discipline of Psychiatry, School of Medicine, Trinity College Dublin, Dublin 2, Ireland; 5 Institute of Psychiatry, King's College London, London SE5 8AF, UK; 6 Department of Psychiatry, Université de Montreal, CHU Ste Justine Hospital, Montréal, Québec, Canada H3C 3J7; 7 Department of Cognitive and Clinical Neuroscience, Central Institute of Mental Health, Medical Faculty Mannheim, Heidelberg University, 68159 Mannheim, Germany; 8 Commissariat à l'Energie Atomique et aux Energies Alternatives, 14 CEA, DSV, I2BM, Neurospin bat 145, 91191 Gif-sur-Yvette, France; 9 Institute of Neuroscience, Trinity College Dublin, Dublin 2, Ireland; 10 Departments of Psychiatry and Psychology, University of Vermont, Burlington, Vermont 05401, USA; 11 School of Physics and Astronomy, University of Nottingham, Nottinghamshire NG7 2RD, UK; 12 Department of Psychiatry and Psychotherapy, Campus Charité Mitte, Charité—Universitätsmedizin Berlin, 10117 Berlin, Germany; 13 Physikalisch-Technische Bundesanstalt (PTB), 10587 Berlin, Germany; 14 Department of Addictive Behaviour and Addiction Medicine, Central Institute of Mental Health, Medical Faculty Mannheim, Heidelberg University, 68159 Mannheim, Germany; 15 Institut National de la Santé et de la Recherche Médicale, INSERM Unit 1000 ‘Imaging & Psychiatry', University Paris-Sud, 91400 Orsay, France; 16 Maison de Solenn, APHP Hôpital Cochin, University Paris Descartes, 75006 Paris, France; 17 McGill University and Genome Quebec Innovation Centre, Montréal, Québec, Canada H3A 1A4; 18 Rotman Research Institute, University of Toronto, Toronto, Ontario, Canada M5S 3E6; 19 School of Psychology, University of Nottingham, Nottingham, Nottinghamshire NG7 2RD, UK; 20 Montreal Neurological Institute, McGill University, Montreal, Quebec, Canada H3A 2B4; 21 The Hospital for Sick Children, University of Toronto, Toronto, Ontario Canada, M5G 1X8; 22 Behavioural and Clinical Neurosciences Institute, Department of Experimental Psychology, University of Cambridge, Cambridge CB2 3EB, UK; 23 Department of Psychiatry and Psychotherapy, and Neuroimaging Center, Technische Universität Dresden, 01307 Dresden, Germany; 24 MRC Social, Genetic and Developmental Psychiatry (SGDP) Centre, London SE5 8AF, UK; 25 PERTIMM, 92600 Asnieres-Sur-Seine, France; 26 Department of Psychology, University of Sussex, Falmer BN1 9QH, UK; 27 Warwick University, Coventry CV4 7AL, UK; 28 GABO:Milliarium mbH & Co., KG 80333 Munich, Germany; 29 Deutsches Referenzzentrum fur Ethik, D53113 Bonn, Germany; 30 Delosis, Twickenham, Middlesex TW1 4AE, UK; 31 Scito, F-75020 Paris, France; 32 Centre National de Genotypage, 91057 Evry, France.

## Abstract

Novelty-seeking tendencies in adolescents may promote innovation as well as problematic impulsive behaviour, including drug abuse. Previous research has not clarified whether neural hyper- or hypo-responsiveness to anticipated rewards promotes vulnerability in these individuals. Here we use a longitudinal design to track 144 novelty-seeking adolescents at age 14 and 16 to determine whether neural activity in response to anticipated rewards predicts problematic drug use. We find that diminished BOLD activity in mesolimbic (ventral striatal and midbrain) and prefrontal cortical (dorsolateral prefrontal cortex) regions during reward anticipation at age 14 predicts problematic drug use at age 16. Lower psychometric conscientiousness and steeper discounting of future rewards at age 14 also predicts problematic drug use at age 16, but the neural responses independently predict more variance than psychometric measures. Together, these findings suggest that diminished neural responses to anticipated rewards in novelty-seeking adolescents may increase vulnerability to future problematic drug use.

## Abstract (web summary)

Some adolescents seek novelty, but it is unknown whether the brain circuits underlying this behaviour can be used to predict later, problematic behaviour. Here, authors show that diminished ventral striatal and prefrontal activity in response to anticipated rewards at age 14 in these individuals predicts problematic drug use at age 16.

Individual differences in novelty seeking are associated with impulsive choice (or a preference for smaller but sooner over larger but later rewards)^1^,^2^,^3^. Specifically, novelty-seeking traits in adolescents^4^ can foreshadow later problematic behaviours including excessive drug use^2^,^5^,^6^. Novelty seeking, in general, and impulsive choice, in particular, may recruit distinct neural systems^7^,^8^,^9^ that include a motivational circuit comprising mesolimbic dopamine projections from the ventral tegmental area of the midbrain to the ventral striatum (VS)^10^ as well as a countervailing cognitive control circuit comprising prefrontal cortical (PFC) regions. The balance of activity in these circuits may shift over development, consistent with evidence for earlier development of the motivational circuit than the cognitive control circuit in humans^11^,^12^,^13^,^14^. Since dopaminergic modulation of these circuits can influence both motivation^10^ and cognitive control^15^, delays in the development of these circuits and their relative activity could increase impulsive choice, including drug use^2^.

Theoretical accounts differ, however, with respect to exactly how activity in these motivational and control circuits can influence impulsive choice in adolescents^16^. On the one hand, impulsive choice in adolescents has been attributed to diminished motivation, such that drug abuse may reflect attempts to compensate for motivational deficits^17^,^18^. Support for this account has come from neuroimaging studies, suggesting that adolescents show diminished responses during anticipation of monetary rewards relative to adults^19^,^20^,^21^, which are more pronounced in adolescents with contemporaneous drug use^22^. On the other hand, impulsive behaviour in adolescents has also been attributed to excessive motivation^7^,^23^, which could magnify the impact of received rewards and fuel subsequent impulsive choice^8^,^24^. Support for this countervailing view comes from neuroimaging studies, indicating that adolescents show enhanced responses to monetarily rewarding outcomes relative to adults^7^,^23^,^25^. More recent integrations of these findings can resolve these apparent discrepancies by clarifying that adolescents show both diminished responses during reward anticipation, as well as increased responses to reward outcomes, relative to adults^26^,^27^.

For novelty-seeking adolescents, impulsive choices may confer benefits as well as costs^28^. Although novelty-seeking adolescents have been labelled as ‘reckless', ‘stupid', ‘irrational', ‘callous', ‘lazy' or even ‘violent'^29^, novelty seeking could confer either proximal or distal advantages. For instance, novelty seeking encourages emigration away from relatives (which minimizes inbreeding)^30^, and can facilitate discovery and exploration of new opportunities and behaviours that might prove useful later in life. Novelty seeking may also increase self-esteem when valued by peers, since peer influence increases over adolescence^31^. Finally, novelty seeking can elevate reproductive success in competitive environments in other species^30^ as well as humans, since others might perceive willingness to pursue novel options as a marker of ability^32^. For instance, in business, novelty seeking has been associated with creativity, entrepreneurial initiative and commercial success^33^.

Thus, while novelty-seeking behaviour can both harm and help adolescents, it is currently unclear how or when novelty-seeking traits promote pathology versus promise. In this research, we used a longitudinal design to identify which neural and behavioural factors predispose novelty-seeking adolescents to harmful outcomes specifically related to problematic drug use (PDU). This design could reveal whether functional or structural neural markers at age 14 preceded PDU at age 16. We targeted motivational circuitry using a variant of a well-established neuroimaging task that reliably indexes individual differences in neural activity during reward anticipation (that is, the Monetary Incentive Delay Task)^34^,^35^. Previous research has associated mesolimbic activity during reward anticipation with dopamine release^36^ as well as craving for drugs of abuse^37^. The current longitudinal design allowed us to test whether novelty-seeking adolescents with decreased neural responses during reward anticipation would be more likely to develop PDU (defined as the intake of increased amounts of licit and/or illicit drugs) over 2 years later. This design also afforded a direct comparison of neural versus psychometric predictors of PDU. Based on previous findings implicating blunted neural responses during reward anticipation in adolescents with contemporaneous PDU^22^, we hypothesized that decreased neural responses during reward anticipation might predict eventual PDU in novelty-seeking adolescents, and further, that these neural markers might augment predictions afforded by more conventional psychometric measures.

Consistent with these hypotheses, we find that novelty-seeking adolescents who go on to develop PDU initially show reduced neural activity during reward anticipation (specifically, in the midbrain, VS and dorsolateral prefrontal cortex). These differences in neural activity cannot be accounted for by volumetric changes, and augment (or even exceed) predictions afforded by more conventional psychometric trait measures (specifically, temporal discounting and low conscientiousness). In the future, neural markers of susceptibility to PDU may help researchers and clinicians to better target problematic symptoms and vulnerable individuals for intervention.

## Results

### Sample characteristics

Although the critical predictions focused on novelty-seeking adolescents, we first sought to verify that novelty seeking was associated with PDU. In the target sample of subjects with high novelty-seeking scores (highest 25th percentile: *n*=283), 72 qualified as having PDU (25.4% PDU). Among adolescents in the middle 25–75% of novelty seekers (*n*=552), 102 qualified as having PDU (18.5% PDU), whereas in the lowest 25th percentile of novelty seekers (*n*=255), only 18 qualified as having PDU (7.1% PDU). Incidence percentages thus supported the assumption that novelty-seeking traits appear relevant, but not sufficient, to confer vulnerability to PDU.

### Behavioural and psychometric data

Comparisons also verified that the PDU group showed significantly greater drug-taking scores than the control group at age 16 (*n*=72 controls 9.83±4.66, *n*=72 PDU group 20.24±5.43, *F*(1,142)=152.19, *P*<10^−10^, analysis of variance (ANOVA)), even though these differences were not evident at age 14 (*n*=72 controls 6.33±3.04, *n*=72 PDU group 6.93±4.97, *F*(1,142)=0.76, *P*=0.39, ANOVA; group (PDU versus control) by time point (age 14 versus age 16) interaction *F*(1,284)=81.33, *P*<2 × 10^−16^, ANOVA). The PDU and control groups did not significantly differ with respect to pubertal status, age, gender, intelligence, novelty-seeking score, risk taking (CGT) or overall hit rate and reaction times in the Monetary Incentive Delay (MID) task (with the exception of the no gain condition; see Table 1) at age 14. The PDU group did, however, show steeper discounting of future rewards (log(discount rate); *n*=72 controls: −4.55±1.38, *n*=72 PDU: –3.99±1.53, *F*(1,142)=5.23, *P*=0.024; ANOVA) and scored lower in conscientiousness (*n*=72 controls: 25.44±6.20, *n*=72 PDU: 23.13±6.13, *F*(1,142)=5.09, *P*=0.026, ANOVA; see Table 1) at age 14.

### Functional neuroimaging data

**Figure 1:**
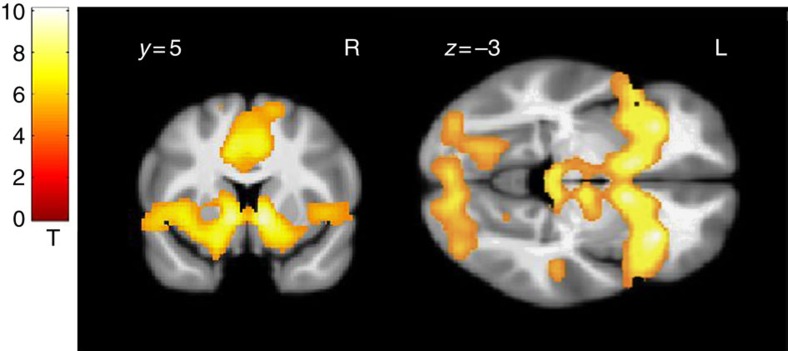
fMRI activity during anticipation of large versus small gains for control and PDU subjects combined (*n*=144). Overlaid on a mean structural magnetic resonance scan showing a coronal (left) and an axial (right) section, activation display threshold is *P*<0.05 (whole brain corrected, *t*-test).

A first confirmatory voxel-wise analysis contrasted whole-brain activity during large versus small gain anticipation across both groups to verify main effects of reward anticipation at age 14. Across groups, large versus small gain anticipation elicited expected increases in activity in mesolimbic regions including the VS (*n*=144; peak *x*, *y*, *z*: 11, 5, −5 mm, *Z*=7.8, *P*=1.8E−13, corrected, *t*-test; −11, 5, −5 mm, *Z*=7.3, *P*=8.8E−12, corrected) and midbrain (peak *x*, *y*, *z*: 6, −25, −12 mm, *Z*=5.3, *P*=1.7E−6, corrected; −8, −24, −9 mm, *Z*=4.8, *P*=2.1E−5, corrected *t*-test; Fig. 1).

**Figure 2:**
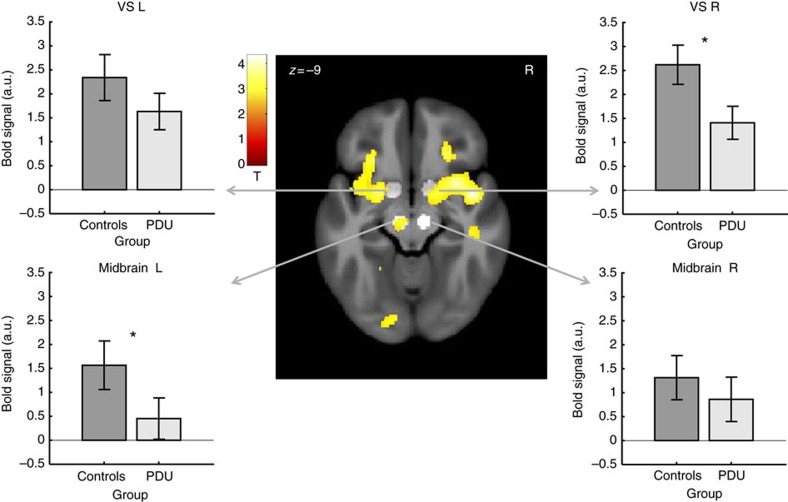
Subcortical brain activity in anticipation of large versus small gains for control subjects (*n*=72) versus problematic drug users (*n*=72). The PDU group showed decreased activation in bilateral ventral striatum (left, right) and midbrain (bottom). Overlaid on a mean structural magnetic resonance scan, activation display threshold is *P*<0.005 (uncorrected, *t*-test). Highlighted areas indicate volumes of interest in the ventral striatum (VS foci: ±14, 8, –8) and midbrain (VTA foci: ±9, –15, –15). Error bars=±s.e.m. *Significant at threshold of *P*<0.0083 uncorrected or *P*<0.05 corrected (*n*=144, *t*-test).

**Figure 3:**
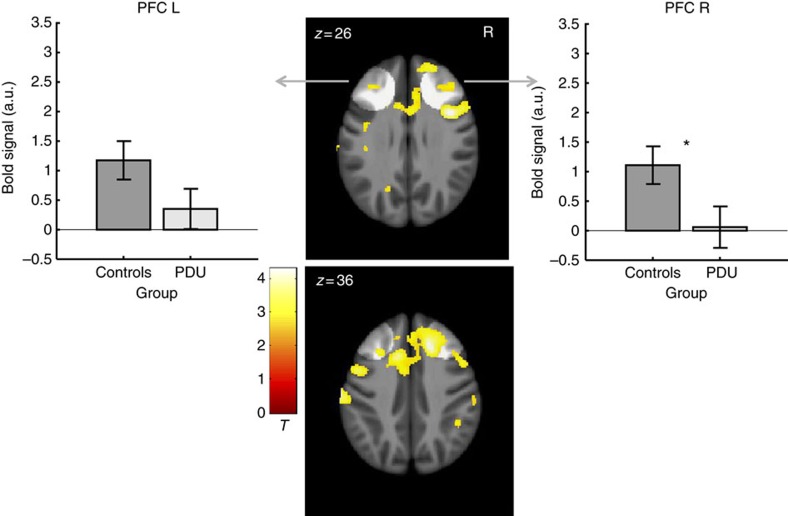
Cortical brain activity in anticipation of large versus small gains for control subjects (*n*=72) versus problematic drug users (*n*=72). The PDU group showed decreased activation in the right dorsolateral prefrontal cortex (for VOI-based statistics, see Table 2). Overlaid on a mean structural magnetic resonance scan, activation display threshold is *P*<0.005 (uncorrected, *t*-test). Highlighted areas indicate volumes of interest in the dorsolateral prefrontal cortex (PFC foci: ±35, 36, 32). Error bars=±s.e.m. *Significant at threshold of *P*<0.0083 uncorrected or *P*<0.05 corrected (n=144, *t*-test).

The second targeted analysis contrasted activity in six predefined volumes of interest (see Methods) during large versus small gain anticipation in the PDU versus control groups at age 14 (Table 2; Figs 2 and 3; Supplementary Fig. 1). This analysis revealed significant group differences in activity in the right VS (*n*=144; *t*(126)=–2.66, *P*=0.004, uncorrected/*P*=0.027, corrected, *t*-test), the left midbrain (*n*=144; *t*(126)=–2.69, *P*=0.004, uncorrected/*P*=0.024, corrected, *t*-test) and right dorsolateral prefrontal cortex (*n*=144; *t*(126)=–2.48, *P*=0.007, uncorrected/*P*=0.044, corrected, *t*-test). Although this targeted analysis focused on high novelty-seeking adolescents, based on their documented vulnerability to future PDU, we further examined ventral striatal activity in subjects who scored in the middle quartiles and lower quartile on novelty-seeking traits. Decreased ventral striatal activity in the PDU group was only evident, however, in high novelty-seeking adolescents (Supplementary Figs 2 and 3).

A third exploratory voxel-wise analysis contrasted whole-brain activity during large versus small gain anticipation in the PDU versus control groups at age 14. Consistent with targeted findings, this analysis revealed group differences in activity in foci located in the bilateral VS (*n*=144; peak *x*, *y*, *z*: 15, −3, −9 mm, *Z*=–3.2, *P*=7.9E−4, uncorrected, *t*-test; peak *x*, *y*, *z*: −18, 0, −6 mm, Z=–2.9; *P*=0.002, uncorrected, *t*-test), left midbrain (peak *x*, *y*, *z*: −8, −21, −9 mm, Z=–3.1, *P*=9.0E−4, uncorrected, *t*-test), and right dorsolateral prefrontal cortex (peak *x*, *y*, *z*: 37, 5, 25 mm, *Z*=–4.2, *P*=1.7E−5, uncorrected; *x*, *y*, *z*: 18, 21, 36 mm, *Z*=–4.1, *P*=2.2E-5, uncorrected, *t*-test; Figs 2 and 3).

### Structural neuroimaging data

**Figure 4:**
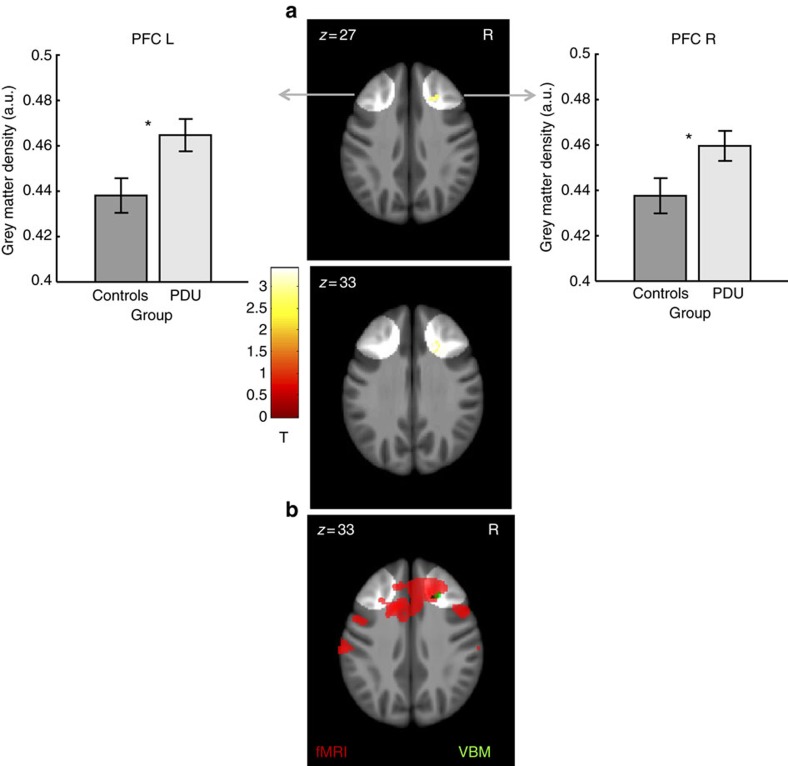
Cortical differences in grey matter volume for control subjects (*n*=72) versus prospective problematic drug users (PDU) (*n*=72). (**a**) Increased grey matter density was observed for the PDU group in the right dorsolateral prefrontal cortex. (**b**) The location of increased grey matter density (green) lies adjacent to reduced activation in the Monetary Incentive Delay (MID) task for the prospective problematic drug users (red). Overlaid on a mean structural magnetic resonance scale, volumetric display threshold is *P*<0.005 (uncorrected, *t*-test). Highlighted areas indicate volumes of interest in the prefrontal cortex. Error bars=±s.e.m. *Significant at threshold of *P*<0.0083 uncorrected or *P*<0.05 corrected (*n*=144, *t*-test).

Reduced neural activity that precedes PDU could result from abnormal neural function, abnormal structure or both^38^. Volume of interest analysis of voxel-based morphometry indices of grey matter density in the same six volumes used for functional comparisons revealed significant group differences after correcting for multiple comparisons (Table 3; Fig. 4a; Supplementary Fig. 4). Specifically, at age 14, high novelty seekers who eventually developed PDU showed increased grey matter density in the left VS (*n*=144; *t*(126)=2.51, *P*=0.007, uncorrected/*P*=0.040, corrected, *t*-test), the left midbrain (*n*=144; *t*(126)=2.95, *P*=0.002, uncorrected/*P*=0.011, corrected, *t*-test) and bilateral dorsolateral prefrontal cortex (right: *n*=144; *t*(126)=3.62, *P*=0.001, uncorrected/*P*=0.001 corrected; left: *t*(126)=3.76, *P*=0.001, uncorrected/*P*=0.001, corrected, *t*-test). The dorsolateral prefrontal region that showed structural differences showed some overlap with regions that showed group differences in functional activity (Fig. 4b).

### Behavioural and neural prediction of PDU

To compare the ability of psychological and neural variables of interest to predict the development of PDU, we further implemented a series of logistic regression models using statistically relevant psychometric and neural variables acquired at age 14 to predict PDU at age 16. Model comparison revealed that a model combining neural (activation in VS and dlPFC) with psychological variables (temporal discounting and Neuroticism-Extraversion-Openness Five-Factor Inventory conscientiousness) at age 14 best-predicted PDU at age 16 (*n*=144, Akaike Information criterion (AIC)=176, pseudo *R*^2^=0.20, logistic regression). Interestingly, the next most-predictive model included only neural variables (*n*=144, AIC=183, pseudo *R*^2^=0.15, logistic regression), followed by the model that included only psychological variables (*n*=144, AIC=195, pseudo *R*^2^=0.07, logistic regression). These findings suggest that neural and psychological variables may account for independent variance in predicting PDU. Performing a formal classification using a linear support vector machine with threefold cross-validation implied that model predictions should generalize to other samples, and that classification accuracy was similar for the combined (neural and psychological) model (66% out of sample) and the model containing only neural variables (65% out of sample), but lower for the model containing only psychological variables (55% out of sample; Table 4).

## Discussion

To identify factors that confer vulnerability to PDU, we longitudinally characterized and tracked a large sample of novelty-seeking adolescents. We then compared individuals at age 14 who subsequently developed PDU at age 16 with those who did not. Importantly, these groups were carefully matched at age 14 on a range of relevant variables, including drug use. Individuals who later transitioned to PDU showed decreased right ventral striatal, left midbrain and right dorsolateral prefrontal cortex activity during anticipation of large versus small gains at age 14. Consistent with greater impulsivity, these vulnerable individuals also showed steeper discounting of future rewards and lower conscientiousness scores at age 14. Notably, comparison of neural and psychological measures revealed that the neural markers predicted PDU as well as or better than the psychological variables.

Consistent with the primary prediction, novelty-seeking subjects with less ventral striatal activity during reward anticipation at age 14 were more likely to develop PDU at age 16. Reduced ventral striatal activity during gain anticipation has previously been observed in cross-sectional studies of substance abuse^39^ and other addictive behaviours^40^, and coheres with non-human primate research, suggesting that repeated drug intake can reduce activity in the VS^41^. Cross-sectional research, however, cannot clarify whether diminished neural responses to gain anticipation precede or result from substance abuse. Some relevant evidence, however, comes from longitudinal studies of animals. For instance, one study implied that reduced dopamine D2 receptor availability in the striatum (assessed with positron emission tomography or PET) foreshadowed increased drug intake in primates^41^. Another study of rodents bred for impulsivity also indicated that reduced D2/D3 receptor availability in the striatum preceded increased drug intake^42^. This animal research highlights a critical role for longitudinal designs in clarifying causal pathways to substance abuse in humans. Consistent with animal results, the current findings demonstrate in a longitudinal sample of novelty-seeking adolescents that reduced activation of the VS and the midbrain at age 14 precedes PDU at age 16, thus implying that reduced recruitment of the mesolimbic circuit not only results from, but also can precede and predict PDU.

Theorists have linked reduced activity in the mesolimbic circuit to blunted motivation for reward^17^,^18^,^30^. Since organisms typically seek to increase states associated with positive outcomes^10^, individuals with blunted neural responses during reward anticipation may require the promise of stronger rewards (for example, drugs of abuse) to elicit comparable levels of motivation. The reduced ventral striatal activation at age 14 observed in novelty-seeking adolescents at risk for PDU does not necessarily negate findings, suggesting that drug use may also reciprocally decrease ventral striatal activity^41^. Instead, in combination with previously noted cross-sectional observations of reduced mesolimbic activity associated with addictive behaviour^39^,^40^, the present longitudinal findings raise the possibility of a vicious cycle in which novelty-seeking individuals with less responsive mesolimbic circuits seek increased exposure to drugs of abuse, which can further blunt mesolimbic responsiveness, and so maintain addiction^2^.

In neuroimaging tasks that elicit reward anticipation (for example, the Monetary Incentive Delay Task), researchers have reported that subjects show increased ventral striatal activity during the anticipation of large gains in comparison with small gains, no gains and even comparable losses^34^,^43^. As in a previous cross-sectional study of adolescent smokers^22^, at-risk adolescents showed reduced ventral striatal activity during gain anticipation, but not in response to gain outcomes. Electrophysiological recordings in primates suggest that the firing of midbrain dopaminergic neurons increases proportional to anticipated gain magnitude^44^, but that firing in response to gain outcomes instead reflects the inverse likelihood of previously anticipated gain (that is, the surprisingness of the gain outcome)^45^. More recently, optogenetic functional magnetic resonance imaging (fMRI) research on rats indicated that phasic optogenetic stimulation of midbrain dopamine neurons increases fMRI activity in the striatum^46^. Thus, the present findings are consistent with an account in which ventral striatal activity during reward anticipation reflects phasic increases in dopamine firing and consequent release in the VS. More support for this account comes from a human study that combined the MID task with fMRI as well as [11C]raclopride PET to demonstrate that individuals who showed more fMRI activity during gain anticipation also showed more PET evidence of dopamine release to gain cues in the VS. Correspondence across imaging modalities was not evident, however, in striatal responses to gain outcomes^36^. In the present study, midbrain activity correlated robustly with ventral striatal activity during reward anticipation, and blunting of this anticipatory response predicted subsequent PDU at age 16 (Supplementary Table 1).

Although neuroimaging tasks less reliably elicit dorsolateral PFC activity than ventral striatal activity during reward anticipation, dlPFC regions also showed reduced activity during reward anticipation in novelty-seeking adolescents who went on to develop PDU. Theorists have posited that impulsive adolescent choice may stem from imbalances in the activity of rapidly developing mesolimbic motivational circuits versus more slowly maturing prefrontal control circuits^8^,^9^. dlPFC activity has specifically been associated with planning, behavioural control and goal implementation^47^,^48^. More extensive longitudinal assessments might clarify whether reduced prefrontal functional activity reflects a developmental delay or a lasting deficit in adolescents at risk for PDU. Since ventral striatal regions connect to the prefrontal cortex through thalamic relays, which then reciprocally modulate the striatum^10^, additional research might also clarify whether reduced ventral striatal activity precedes reduced prefrontal activity or the opposite. Since PDUrs showed higher (rather than lower) grey matter density in dorsolateral prefrontal cortex regions, the observed decreases in functional activity could not be attributed to decreased structural grey matter integrity (for example, as in the case of partial voluming). Observed increases in dorsolateral prefrontal grey matter density are consistent, however, with the notion of a structural developmental delay in prospective problematic drug users at age 14, since developmental studies suggest that PFC thickness continually decreases over adolescence^11^,^12^, possibly as a result of synaptic pruning. Therefore, relatively greater prefrontal grey matter density might reflect maturational delays in novelty-seeking adolescents that presage PDU.

Psychometric and behavioural measures have historically offered powerful tools for assessing individual differences in consideration of future rewards and long-term goals. For instance, measures of temporal discounting index a preference for smaller sooner rewards over larger later rewards. Low temporal discounting powerfully predicts future educational and economic success^49^,^50^, whereas high temporal discounting has instead been associated with addictive behaviour^22^,^51^,^52^,^53^. Consistent with this cross-sectional evidence, the present longitudinal findings indicate that high temporal discounting at age 14 was associated with PDU at age 16 in high novelty-seeking adolescents. Measures of conscientiousness index the tendency to follow socially prescribed norms for impulse control^54^, whereas low conscientiousness has been associated with a wide range of addictive behaviours (including tobacco, alcohol and drug use)^55^. The present findings additionally indicate that low conscientiousness at age 14 was associated with PDU at age 16 in high novelty-seeking adolescents. While both high temporal discounting and low conscientiousness have been linked to compromised PFC function^54^,^56^,^57^,^58^, direct model comparisons indicated that reduced ventral striatal and dorsolateral PFC activity during reward anticipation might uniquely contribute to predictions of PDU in novelty-seeking adolescents—above and beyond contributions from these relevant psychometric measures.

Despite strengths of the study design in combining validated neuroimaging probes with substantial matched longitudinal samples^59^, the design also has some limitations. For instance, cutoff criteria for PDU necessarily depend upon specific substances under consideration. In contrast to alcohol and cigarette consumption, in which a score compatible with daily use represented the cutoff, the threshold for other illicit drugs was instead defined based on lifetime use. For this longitudinal sample, low thresholds were adopted (particularly for illicit drugs such as crack, cocaine and narcotics), relative to other studies of early use^60^. Only a few adolescents qualified for PDU at age 16 with respect to use of illicit drugs (Supplementary Figs 5 and 6), whereas most instead qualified based on the use of licit drugs (for example, alcohol, cigarette or cannabis). The validity of the adopted criteria was supported, however, by the fact that the criteria predicted future PDU. Specifically, the percentage of adolescents qualifying for PDU at age 16 was highest for the top quarter of novelty seekers (25.4% PDU), lower for the middle two quarters of novelty seekers (18.5% PDU) and lowest for the bottom quarter of novelty seekers (7.1% PDU). Based on previous research, we adopted a binary threshold criterion for PDU instead of a continuous outcome measure. This classification skirted correlational assumptions that the total amount of substance use maps linearly onto vulnerability. Such a correlational design might assume, for instance, that an individual who uses cigarettes, cannabis and alcohol should show a threefold difference in brain activity in predicted neural targets (for example, the VS) relative to an individual who uses only cannabis. These linear assumptions stand in contrast to the notion that substance abuse may reflect the expression of an addictive syndrome^61^. Consistent with such a categorical distinction, only in the high novelty-seeking group did blunted ventral striatal activity clearly foreshadow later PDU (Supplementary Fig. 2). Further, while stressors and related negative arousal may also potentiate impulsive behaviours including substance abuse in adolescents^62^,^63^, the neuroimaging task employed in this study elicited gain but not loss anticipation, and so was primarily optimized to probe neural responses during anticipation of reward. Future research using neuroimaging probes that elicit anticipation of punishment might better probe links between negative arousal and adolescent vulnerability to substance abuse.

In conclusion, these longitudinal findings in novelty-seeking adolescents demonstrate that diminished mesolimbic reward motivation along with impaired prefrontal control may confer risk for future PDU. Importantly, these findings suggest that high novelty seeking alone does not necessarily lead to PDU, and that neuroimaging measures may augment psychometric measures in identifying vulnerability. Rather than limiting developmental possibilities^29^, these findings may help clinicians to visualize modifiable markers that can eventually be therapeutically targeted to prevent vulnerability or even to promote flourishing as novelty seekers transition from adolescence to adulthood^64^.

## Methods

### Subjects

Data for this study came from the IMAGEN project^65^, and were collected at multiple sites across Europe. At age 14, a large cohort of adolescents completed self-report and interview measures, in addition to structural and fMRI scans. Parental report measures were also collected for some constructs. Local ethics research committees approved the study at each site. On the day of assessment, written consent was obtained from each parent or guardian, and verbal assent was obtained from each adolescent. Further details on recruitment, standardized instructions for administration of psychometric and cognitive behavioural measures, and other procedures are described in the Standard Operating Procedures for the IMAGEN project (http://www.imagen-europe.com/en/Publications_and_SOP.php). Subjects were included in the current study if they had valid data for all measures including the initial assessment at age 14 and follow-up at age 16 (see below). Based on these criteria, at the time of analysis, complete data were available for 1,090 adolescents.

**Figure 5:**
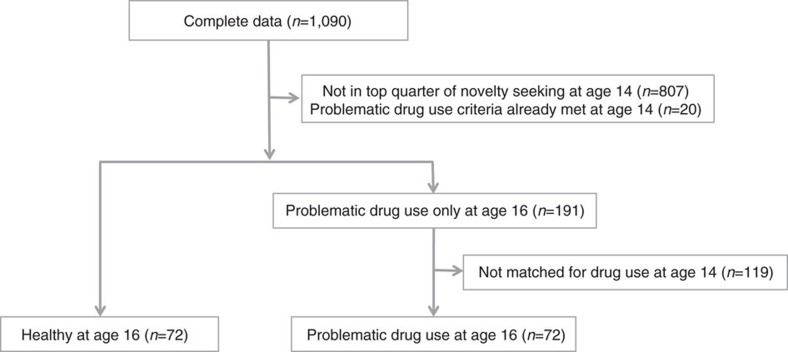
Experimental design diagram depicting subject selection procedure. Out of 1090 subjects with full datasets, the top quarter of novelty seekers who had not already met criteria for problematic drug use at age 14 were selected. Those who showed problematic drug use at age 16 were matched with those who did not with respect to drug use at age 14 (*n*=72 per group, 144 total).

Novelty seeking in this sample was initially assessed with the Novelty Seeking subscale of the Temperament and Character Inventory—Revised^66^. From the original sample of 1,090 with full data sets, individuals scoring in the top 25th percentile (*n*=283) of novelty seeking at the initial assessment at age 14 were selected. Based on the criteria described below, these subjects were then classified either as having PDU either at age 14 (excluded; *n*=20) or at age 16 (PDU group; *n*=72) or as not having PDU at either ages 14 or 16 (control group; *n*=191). Since we aimed to classify whether neural markers predict or result from drug use, we sought to directly compare the PDU group to the control group without statistically significant differences in PDU at age 14. Thus, we further matched both groups with respect to size and average drug intake at age 14 (as defined by each individual's total drug intake score, described below). This procedure yielded 72 subjects in the PDU group and 72 matched subjects in the control group (Table 1; Fig. 5).

### PDU criteria

PDU was operationally defined based on measures according to the European School Survey Project on Alcohol and Other Drugs (ESPAD)^67^. Unlike traditional clinical instruments (for example, the Diagnostic and Statistical Manual of Mental Disorders, version 5) these measures provided a preclinical index of drug use at ages 14 and 16. Thus, cutoff criteria for PDU were defined to capture problematic use of various legal or illicit drugs. With respect to legal drugs (alcohol and cigarettes), a threshold was set that indicated daily use. In particular, a score of 3 or higher on smoking (0: ‘Not at all', 1: ‘Less than 1 cigarette per week', 2: ‘Less than 1 cigarette per day', 3: ‘1–5 cigarettes per day', 4: ‘6–10 cigarettes per day', 5: ‘11–20 cigarettes per day', 6: ‘More than 20 cigarettes per day') and a score of 5 or higher on alcohol consumption (0: ‘0 drinks per month', 1: ‘1–2 drinks per month', 2: ‘3–5 drinks per month', 3: ‘6–9', 4: ‘10–19', 5: ‘20–39' and 6: ‘40 or more') within the last 30 days were defined as PDU.

With respect to illicit drugs, PDU thresholds were based on lifetime use. Apart from cannabis, where the threshold was set to 39 lifetime occasions, the threshold for other drugs (glue, tranquilizers, amphetamine, lysergic acid diethylamide (LSD), hallucinogenic mushrooms, 3,4 methylenedioxymethamphetamine (MDMA), ketamine or liquid ecstasy) was set to 3–5 occasions or more. Use of a lifetime score and therefore a lower threshold criterion for these drugs was justified by the fact that early use of these drugs robustly predicts PDU later in life (for example, a threefold risk after early cannabis use^60^). Finally, the threshold for illicit drugs (for example, crack, cocaine, heroin and narcotics) was set to 1–2 or more occasions (Supplementary Figs 5 and 6 depict the distribution of scores for both groups at age 14 and 16, and cutoffs for each substance). None of the subjects in either group fell above the threshold for use of any substance at age 14 (Supplementary Fig. 5). Furthermore, most subjects were classified as PDU by means of daily cigarette use, followed by alcohol and cannabis—only a few were classified as PDU based on their use of other illicit drugs (for example, MDMA and amphetamines; Supplementary Figs 5 and 6).

### Personality and psychopathology measures

Novelty seeking was assessed using a subscale of the Temperament and Character Inventory—Revised^66^. Dimensions of personality were assessed using the 60-item Neuroticism-Extraversion-Openness Five-Factor Inventory, which indexes dimensions of Extraversion, Agreeableness, Conscientiousness, Neuroticism, and Openness to Experience, as described by the Five-Factor Model of personality^68^. Adolescent psychiatric symptoms and their impact were assessed with the Development and Well-Being Assessment, which generates probabilities that individuals qualify for Diagnostic and Statistical Manual of Mental Disorders (version 4) psychiatric diagnoses^69^.

### Cognitive measures

Subjects completed a version of the Wechsler Intelligence Scale for Children-IV^70^, which included the Perceptual Reasoning, Matrix Reasoning and Similarities, and Vocabulary scales to index intelligence. The Monetary-Choice Questionnaire (MCQ)^51^ was administered to assess delay discounting—or individual differences in the tendency to choose sooner but smaller over later but larger rewards (this index correlates well with more precise but also more time-consuming measures^71^). Subjects also completed the Cambridge Gambling Task (CGT)^72^, as a behavioural measure of risk seeking.

### Demographics

The Puberty Development Scale^73^ assessed each subject's pubertal status. Socioeconomic status scores were assessed using a composite score that indexed the weighted sum of the following variables: Mother's Education Score, Father's Education Score, Family Stress Unemployment Score, Financial Difficulties Score, Home Inadequacy Score, Neighborhood Score, Financial Crisis Score, Mother Employed Score, Father Employed Score. Negative (that is, high risk) scores were reverse-coded.

### Functional neuroimaging data acquisition and analysis

**Figure 6:**
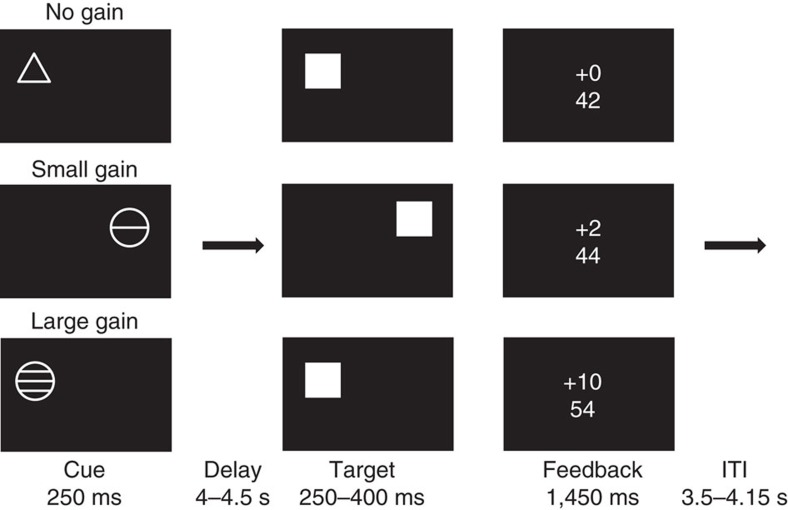
Adapted Monetary Incentive Delay (MID) task trial structure. An initial cue signalled potential gain for each trial (no gain: 0 points; small gain: 2 points; or large gain: 10 points). After a variable delay, a target briefly appeared. Responding during target display yielded the indicated gain, whereas late or early responses yielded no gain. Target durations adapted to approximate a 66% hit rate for each subject^34^.

The task used to probe neural activity during reward anticipation was a modified version of the MID task^34^, which required subjects to respond after seeing a cue to a briefly presented target by pressing one of two buttons as rapidly as possible to indicate whether the target appeared on the left or the right side of the screen (Fig. 6). If subjects responded while the target was on the screen, they received points, but if they responded before or after the target's disappearance, they received no points. Cues signalled each trial's onset, and reliably indicated the position of the target as well as the number of points to be awarded for a successful response. Cues took one of three forms: a triangle indicated no points (‘No Gain'), a circle with one line indicated 2 points (‘Small Gain') and a circle with three lines indicated 10 points (‘Large Gain') at stake. Behavioural data from this modified MID task included the proportion of hits on gain trials and hit reaction times.

At all sites, scanning was performed with 3 T whole-body magnetic resonance scanners produced by a variety of manufacturers (Siemens, Philips, General Electric and Bruker). For functional imaging, we acquired 300 volumes with 40 slices in descending order (2.4 mm slice thickness with 1 mm gap) using a gradient-echo T2*-weighted pulse sequence (EPI). The time to repetition for volume acquisition was set to 2,200 ms and the time to echo to 30 ms. In-plane resolution was 64 × 64 with a field of view of 220 × 220 mm. The plane of acquisition was tilted to parallel the anterior–posterior commissure line. For anatomical reference, a three-dimensional magnetization prepared gradient-echo sequence of the whole brain was obtained with time to repetition of 6.8 ms and a time to echo of 3.2 ms. These imaging parameters were chosen to ensure comparability of data across different scanners. Further details of the image acquisition protocols and quality control procedures have been described previously, including an extensive period of standardization across magnetic resonance scanners^65^.

Image preprocessing and analyses were performed with SPM8 and SPM12 software (Wellcome Trust Centre for Neuroimaging, London). For structural preprocessing, we normalized individually segmented T1-weighted scans to a template generated by the first 552 adolescents in the sample^22^,^38^ using the DARTEL toolbox^74^ as implemented in SPM8. For fMRI whole-brain analyses, single-subject echo-planar images were coregistered with their associated T1-weighted structural images. Functional images were then realigned and resliced to the first volume. Single-subject statistical models analysed the resliced data using the following regressors: (1) anticipation of large gain; (2) anticipation of small gain; (3) anticipation of no gain; (4) feedback indicating large gain; (5) feedback indicating small gain; (6) and feedback indicating no gain. Each regressor was defined separately for successful (that is, ‘hits') as well as unsuccessful (that is, ‘misses') response trials. Thus, each model included a total of 12 orthogonal regressors. Trials in which subjects failed to respond were modelled similarly but separately as error trials. Rigid body movement parameters from the realignment procedure were included as six additional covariates. Next, contrast images of the parameter estimates were created for each subject. The present analyses focused on the gain anticipation phase. Analyses specifically contrasted neural responses during anticipation of large versus small gain, since motor responses did not differ across incentivized conditions between groups (but were slightly lower in vulnerable subjects for no gain trials; Table 1). Single-subject contrast images were created by applying the DARTEL deformations to the contrast images, which were subsequently smoothed with a Gaussian kernel of 8 mm full width at half maximum. Normalized and smoothed single-subject contrast images were then entered into a second-level random-effects analysis (two-sample *t*-test contrasting PDU subjects versus controls). Although both groups were matched for gender, pubertal status, intelligence estimate, ESPAD composite score, novelty-seeking score and scanning site at the first assessment, we included those variables as covariates to account for residual variance between and within groups. For whole-brain analyses, which were primarily intended to verify main effects of the neuroimaging task across groups and group differences in predicted regions, the threshold was set to *P*<0.05, corrected for multiple comparisons.

For targeted analyses that tested the critical hypotheses, bilateral ventral striatal spherical (12 mm diameter) volumes of interest (VOIs) were centred on Montreal Neurological Institute coordinates ±14, 8, –8 (ref. 75), as documented in previous research on reward anticipation^22^,^76^. Similarly, bilateral midbrain spherical (12 mm diameter) VOIs were centred on coordinates ±9, –15, –15 (ref. 77), as documented in previous research on reward anticipation^22^,^76^. Bilateral dorsolateral PFC spherical (40 mm diameter) VOIs were centred on coordinates ±35, 36, 32 (ref. 54), based on previous research on executive control^78^. Tests for associations of activity in these VOIs at age 14 with eventual PDU versus healthy status at age 16 were Bonferroni-corrected for multiple comparisons (*P*<0.05/6=*P*<0.008). In figures, results are displayed at a threshold of *P*<0.005 uncorrected with clusters including at least 10 contiguous voxels and projected onto the mean structural scan of all subjects, but these VOIs are superimposed for visualization purposes (Figs 2, 3, 4). Since the average adolescent brain at age 14 is smaller than the adult brain, we refrained from transforming individual brains into adult MNI space. However, since the predictions came from studies that reported data in MNI space, we estimated the parameters for linear transformation from MNI space to the space of our DARTEL template (that is, *X*=0.892,·*X*_MNI_=−0.008,·*Y*_MNI_=+0.004,·*Z*_MNI_=+0.385; *Y*=0.017,·*X*_MNI_=+0.930,·*Y*_MNI_=+0.025,·*Z*_MNI_=−6.799; *Z*=0.009,·*X*_MNI_=−0.005,·*Y*_MNI_=+0.838,·*Z*_MNI_=−0.093).

### Structural neuroimaging data analysis

Voxel-based morphometry analyses compared spatially normalized structural scans on a voxel-by-voxel basis. Instead of directly comparing image intensity, however, structural scans were segmented into grey and white matter, smoothed, and then the grey matter partition was subjected to a voxel-by-voxel statistical test. Thus, T1 images were segmented using the ‘new segment' routine as implemented in SPM8, then modulated and spatially normalized with DARTEL (see above). Resulting images were smoothed with a Gaussian kernel of 8 mm full width at half maximum. Statistical analyses mirrored those applied to the fMRI contrasts (that is, two-sample *t*-test contrasting PDU subjects versus controls, with gender, pubertal status, intelligence quotient estimate, ESPAD score, novelty-seeking score and scanning site entered as covariates of no interest). Targeted analyses compared grey matter density within the same VOIs that were constructed to compare functional activity.

### Behavioural and neural prediction of PDU

After verifying key psychometric and neural variables at age 14, these variables' relative ability to predict problematic abuse at age 16 was evaluated using a series of logistic regression models that included psychometric variables only, neural variables only (maximum peaks from VOIs), and the combination of psychometric and neural variables. Since data checks of pair-wise correlations revealed that activity in midbrain and bilateral ventral striatal VOIs was highly correlated (Supplementary Fig. 7), and based on previous evidence reliably implicating ventral striatal activity in reward anticipation^76^, we included coefficients for the right ventral striatal VOI in the models. Similarly, since activity in the two dorsolateral prefrontal VOIs was highly correlated, the prefrontal region whose activity was most closely associated with future PDU was included in the models. This initial variable selection averted collinearity and resulting instability that might arise from including highly correlated variables in the same model^79^. Cross-validation analyses verified that these variables could classify future PDU out of sample in each model. Classification was determined using 10-fold cross-validation over fits of a linear support vector machine, with 50% classification representing chance.

### Data availability

Data are available via application to the IMAGEN project (http://www.imagen-europe.com).

## Additional information

**How to cite this article**: Büchel, C. *et al*. Blunted ventral striatal responses to anticipated rewards foreshadow problematic drug use in novelty-seeking adolescents. *Nat. Commun.* **8**, 14140 doi: 10.1038/ncomms14140 (2017).

**Publisher's note**: Springer Nature remains neutral with regard to jurisdictional claims in published maps and institutional affiliations.

## Supplementary Material

Supplementary Information Supplementary Figures and Supplementary Table

## Figures and Tables

**Table 1:** Subject group characteristics and comparisons.

	**Control (*****n*****=72)**	**Problematic drug use (*****n*****=72)**	**Test**	***P*** **value**
*Demographics*				
Age (days)	14.48 (0.40)	14.38 (0.45)	*F*(1,142)=1.82	0.18
Center site	15 21 5 6 8 8 9	9 11 10 10 12 12 8	*χ*^2^(6)=8.95	0.18
PDS score	2.08 (0.28)	2.07 (0.26)	*F*(1,142)=0.10	0.76
Gender (F/M)	45 27	41 31	*χ*^2^(1)=0.46	0.50
Handedness (L/R)	9 63	6 66	*χ*^2^(1)=0.67	0.41
Intelligence*	167.71 (20.09)	171.29 (18.67)	*F*(1,142)=1.23	0.27
Socioeconomic status composite	7.21 (3.37)	7.15 (3.18)	*F*(1,142)=0.01	0.92
ESPAD composite age 14	6.33 (3.04)	6.93 (4.97)	*F*(1,142)=0.76	0.39
ESPAD composite age 16	9.83 (4.66)	20.24 (5.43)	*F*(1,142)=152.19	0.000**
				
*Behavioural measures*				
CGT risk taking	0.56 (0.14)	0.54 (0.14)	*F*(1,142)=0.79	0.38
MCQ discounting (log)	−4.55 (1.38)	−3.99 (1.53)	*F*(1,142)=5.23	0.024*
MID large gain RT	244.87 (26.55)	253.89 (31.70)	*F*(1,142)=3.43	0.07
MID small gain RT	258.53 (35.72)	263.28 (36.33)	*F*(1,142)=0.62	0.43
MID no gain RT	284.18 (44.64)	300.95 (55.75)	*F*(1,142)=3.97	0.048*
MID large gain prob.	0.70 (0.09)	0.69 (0.09)	*F*(1,142)=0.80	0.37
MID small gain prob.	0.70 (0.10)	0.68 (0.10)	*F*(1,142)=1.26	0.26
MID no gain prob.	0.61 (0.11)	0.56 (0.12)	*F*(1,142)=5.96	0.016*
				
*Personality and psychopathology*				
TCI novelty seeking	127.93 (7.04)	127.42 (6.11)	*F*(1,142)=0.22	0.64
NEO neuroticism	21.75 (7.98)	22.24 (8.48)	*F*(1,142)=0.13	0.72
NEO extraversion	33.22 (4.97)	32.15 (5.41)	*F*(1,142)=1.53	0.22
NEO openness	26.67 (5.49)	26.01 (5.82)	*F*(1,142)=0.48	0.49
NEO agreeableness	28.21 (4.61)	26.99 (5.83)	*F*(1,142)=1.95	0.16
NEO conscientiousness	25.44 (6.20)	23.13 (6.13)	*F*(1,142)=5.09	0.026*
DAWBA emotional	65 2 5	63 7 2	*χ*^2^(2)=4.09	0.13
DAWBA behavioural	55 13 4	54 13 5	*χ*^2^(2)=0.12	0.94
DAWBA hyperactive	63 9	64 8	*χ*^2^(1)=0.07	0.80
DAWBA any	47 16 9	47 18 7	*χ*^2^(2)=0.37	0.83

CGT, Cambridge Gambling Task; DAWBA, Development and Well-Being Assessment; ESPAD, European School Survey Project on Alcohol and other Drugs; MCQ, Monetary-Choice Questionnaire; MID, Monetary Incentive Delay Task; NEO, Neuroticism-Extraversion-Openness Five-Factor Inventory; PDS, Pubertal Development Scale; RT, reaction time; TCI, Temperament and Character Inventory -- Revised.Intelligence reflects the sum of these categories of the Wechsler Intelligence Scale for Children (WISC-IV): Similarities, Vocabulary, Block design, Matrix reasoning, Digit span forward.

^*^Significant at uncorrected threshold of *P*<0.05 (*n*=144, *t*-test).**Significant at uncorrected threshold of *P*<0.01 (*n*=144, *t*-test).

**Table 2:** fMRI activity for high versus low gain anticipation contrast (PDU**>**Control).

**Volume of interest (VOI)**	**Right/Left**	***T*** **(126)**	***P*** **(uncorrected)**
Ventral striatum	R	−2.66	0.004*
	L	−1.75	0.042†
			
Midbrain	R	−1.32	0.095
	L	−2.69	0.004*
			
Dorsolateral prefrontal cortex	R	−2.48	0.007*
	L	−2.10	0.019†

^*^Significant at corrected threshold of *P*≤0.0083 (*n*=144, *t*-test corrected for multiple VOIs).

^†^Significant at uncorrected threshold of *P*≤0.05 (*n*=144, *t*-test).

**Table 3:** Structural differences in grey matter density indexed by voxel-based morphometry (PDU>control).

**Volume of interest (VOI)**	**Right/Left**	***T*** **(126)**	***P*** **(uncorrected)**
Ventral striatum	R	2.25	0.013†
	L	2.51	0.007*
			
Midbrain	R	−0.06	>1
	L	2.95	0.002*
			
Dorsolateral Prefrontal Cortex	R	3.62	0.001*
	L	3.75	0.001*

^*^Significant at corrected threshold of *P*<0.0083 (*n*=144, *t*-test corrected for multiple VOIs).

^†^Significant at uncorrected threshold of *P*<0.05 (*n*=144, t-test).

**Table 4:** Logistic regression models of psychological and neural features predicting problematic drug use in novelty-seeking adolescents two years later.

	**Psychological**	**Neural**	**Combined**
Delay discounting	0.28 (0.12)*2.30		0.29 (0.13)**2.23
Conscientiousness	−0.07 (0.03)*−2.27		−0.07 (0.03)*−2.12
R ventral striatum		−0.11 (0.05)*−2.09	−0.12 (0.05)*−2.16
R dorsolateral prefrontal cortex		−0.30 (0.09)**−3.30	−0.30 (0.10)**−3.07
Pseudo *R*^2^ (ML)	0.07	0.15	0.20
AIC	195	183	176
Classification %	58/55	67/65	74/66

AIC, Akaike Information criterion; ML, maximum likelihood.

Statistics are standardized coefficients, followed by standard errors of the mean in parentheses, and Z-scores (*n*=144, significance: *P<0.05; **P<0.01, *t*-test).

Classification was determined using 10-fold cross-validation over fits of a linear support vector machine, with 50% classification representing chance (train/test rates).

## Notes

**The IMAGEN Consortium**

Mercedes Arroyo^23^, Eric Artiges^9^, Semiha Aydin^14^, Christine Bach^15^, Alexis Barbot^9^, Gareth Barker^5^, Ruediger Bruehl^14^, Anna Cattrell^5^, Patrick Constant^26^, Hans Crombag^27^, Katharina Czech^13^, Jeffrey Dalley^23^, Benjamin Decideur^9^, Sylvane Desrivieres^5^, Tahmine Fadai^1^, Mira Fauth-Buhler^15^, Jianfeng Feng^28^, Irinia Filippi^16,17^, Vincent Frouin^9^, Birgit Fuchs^29^, Isabel Gemmeke^13^, Alexander Genauck^13^, Eanna Hanratty^5^, Bert Heinrichs^30^, Nadja Heym^20^, Thomas Hubner^24^, Albrecht Ihlenfeld^14^, Alex Ing^5^, James Ireland^31^, Tianye Jia^5^, Jennifer Jones^4^, Sarah Jurk^24^, Mehri Kaviani^20^, Arno Klaassen^32^, Johann Kruschwitz^13^, Christophe Lalanne^9^, Dirk Lanzerath^30^, Mark Lathrop^33^, Claire Lawrence^20^, Hervé Lemaitre^16,17^, Christine Macare^5^, Catherine Mallik^5^, Adam Mar^23^, Lourdes Martinez-Medina^5^, Eva Mennigen^24^, Fabiana Mesquita de Carvahlo^5^, Xavier Mignon^26^, Sabina Millenet^15^, Ruben Miranda^16,17^, Kathrin Müller^24^, Charlotte Nymberg^5^, Caroline Parchetka^13^, Yolanda Pena-Oliver^27^, Jani Pentilla^16,17^, Jean-Baptiste Poline^9^, Erin Burke Quinlan^5^, Michael Rapp^13^, Stephan Ripke^24^, Tamzin Ripley^27^, Gabriel Robert^5^, John Rogers^31^, Alexander Romanowski^13^, Barbara Ruggeri^5^, Christine Schmäl^15^, Dirk Schmidt^24^, Sophia Schneider^1^, Florian Schubert^14^, Yannick Schwartz^9^, Wolfgang Sommer^15^, Rainer Spanagel^15^, Claudia Speiser^29^, Tade Spranger^30^, Alicia Stedman^20^, Dai Stephens^27^, Nicole Strache^13^, Andreas Ströhle^13^, Maren Struve^15^, Naresh Subramaniam^23^, David Theobald^23^, Nora Vetter^24^, Helene Vulser^16,17^, Katharina Weiss^13^, Robert Whelan^4^, Steve Williams^5^, Bing Xu^5^, Juliana Yacubian^1^, Tao Yu^5^, Veronika Ziesch^24^

^26^PERTIMM, 92600 Asnieres-Sur-Seine, France, ^28^Warwick University, Coventry CV4 7AL, UK, ^29^GABO:milliarium mbH & Co. KG 80333 Munich, Germany, ^30^Deutsches Referenzzentrum fur Ethik, D53113 Bonn, Germany, ^31^Delosis, Twickenham, Middlesex TW1 4AE, UK, ^32^Scito, F-75020 Paris, France, ^33^Centre National de Genotypage, 91057 Evry, France
