# Supplementary material for: Blunted ventral striatal responses to anticipated rewards foreshadow problematic drug use in novelty-seeking adolescents
Source: Nat Commun. 2017 Feb 21;8:14140. doi: 10.1038/ncomms14140 (PMC5321762; doi:10.1038/ncomms14140)
Supplement: Supplementary Information — Supplementary Figures and Supplementary Table [file ncomms14140-s1.pdf]

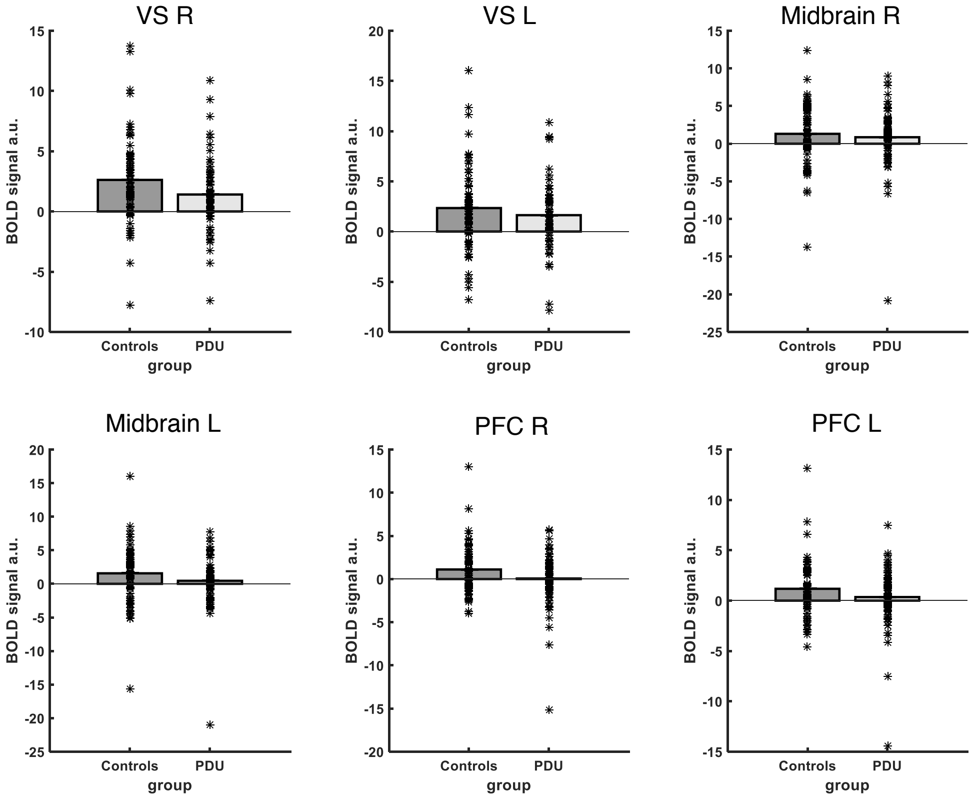

Supplementary Figure 1. Plots overlaid on contrasts of large versus small gain anticipation in all subcortical and cortical volumes of interest for eventual control subjects (n=72) versus problematic drug users (PDU) (n=72; Figures 2 and 3).

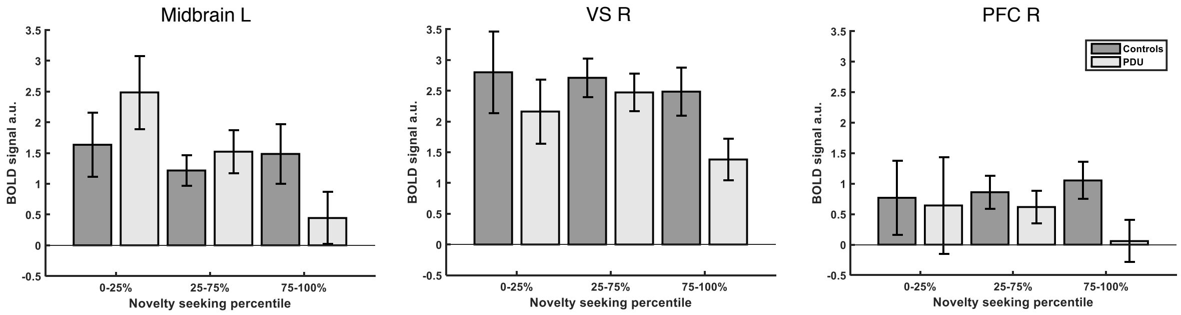

Supplementary Figure 2. Contrasts of large versus small gain anticipation for individuals endorsing low, medium, and high levels of novelty seeking. Depicted regions showed significant activation differences between eventual controls and problematic drug users only in the high novelty-seeking group (Table 2). Plots depict average activation (±SEM) in predefined VOIs (left: left midbrain; middle: right ventral striatum; right: right dorsolateral prefrontal cortex; see Methods for details).

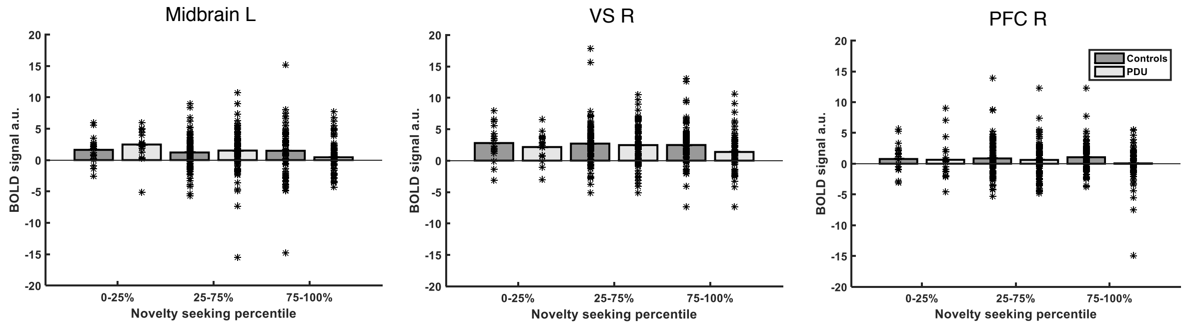

Supplementary Figure 3. Plots overlaid on contrasts of large versus small gain anticipation for volunteers endorsing low, medium, and high levels of novelty seeking. Depicted regions showed significant differences in activation in individuals who went on to develop PDU (n=72) versus not (n=72; Supplementary Figure 2; Table 2).

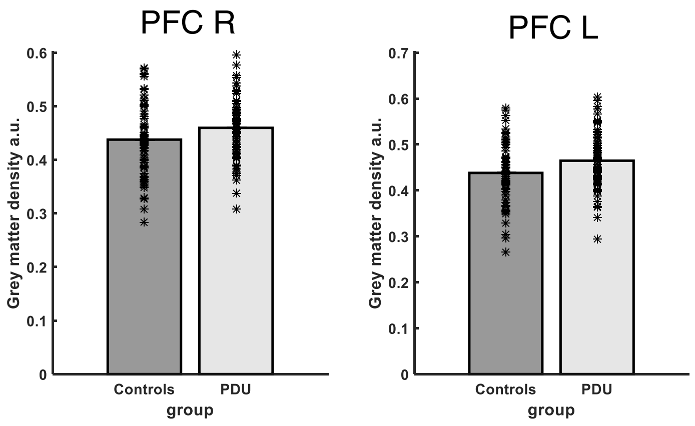

Supplementary Figure 4. Scatterplots of cortical differences in grey matter volume for control subjects (n=72) versus prospective problematic drug users (PDU) (n=72; Figure 5).

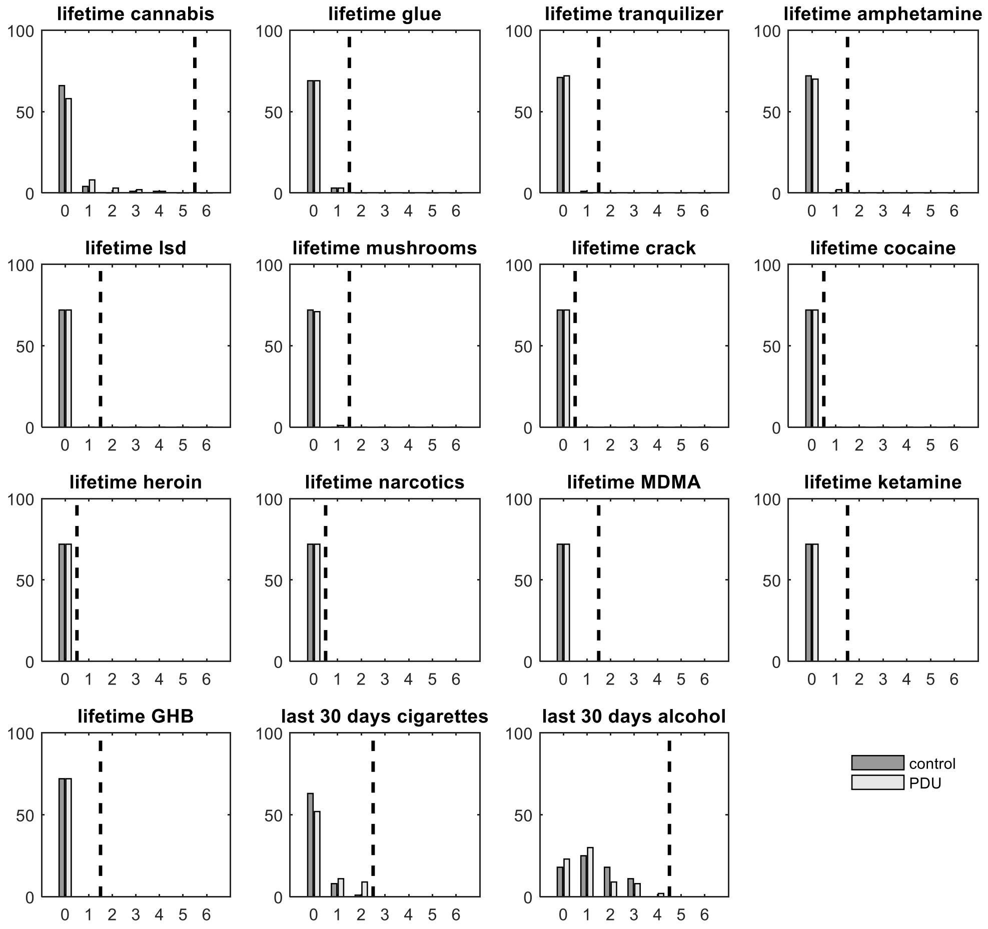

Supplementary Figure 5. Histograms of problematic drug use (ESPAD scores) in control and PDU groups at age 14. The dashed line indicates the predefined threshold for problematic drug use.

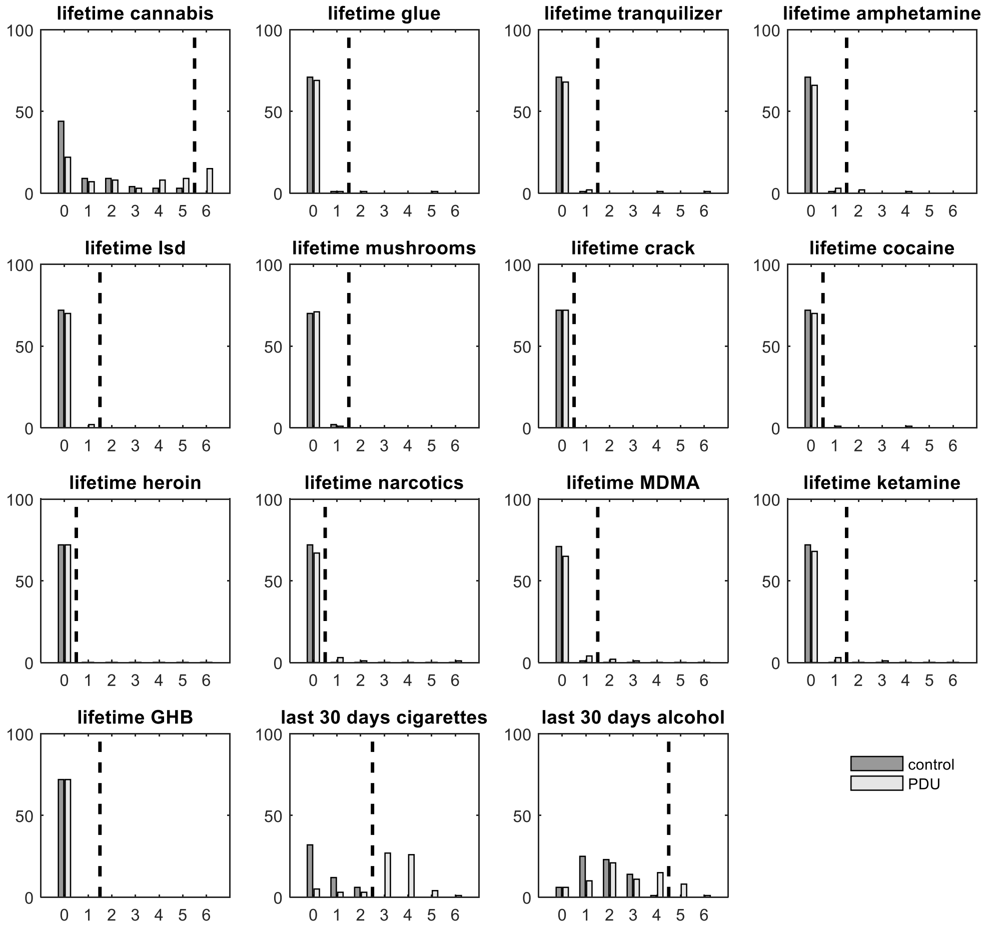

Supplementary Figure 6. Histograms of problematic drug use (ESPAD scores) in control and PDU groups at age 16. The dashed line indicates the predefined threshold for problematic drug use. Subjects above the threshold in any of these categories qualified for our criterion of PDU (most subjects qualified for PDU based on cigarette or alcohol use, followed by cannabis).

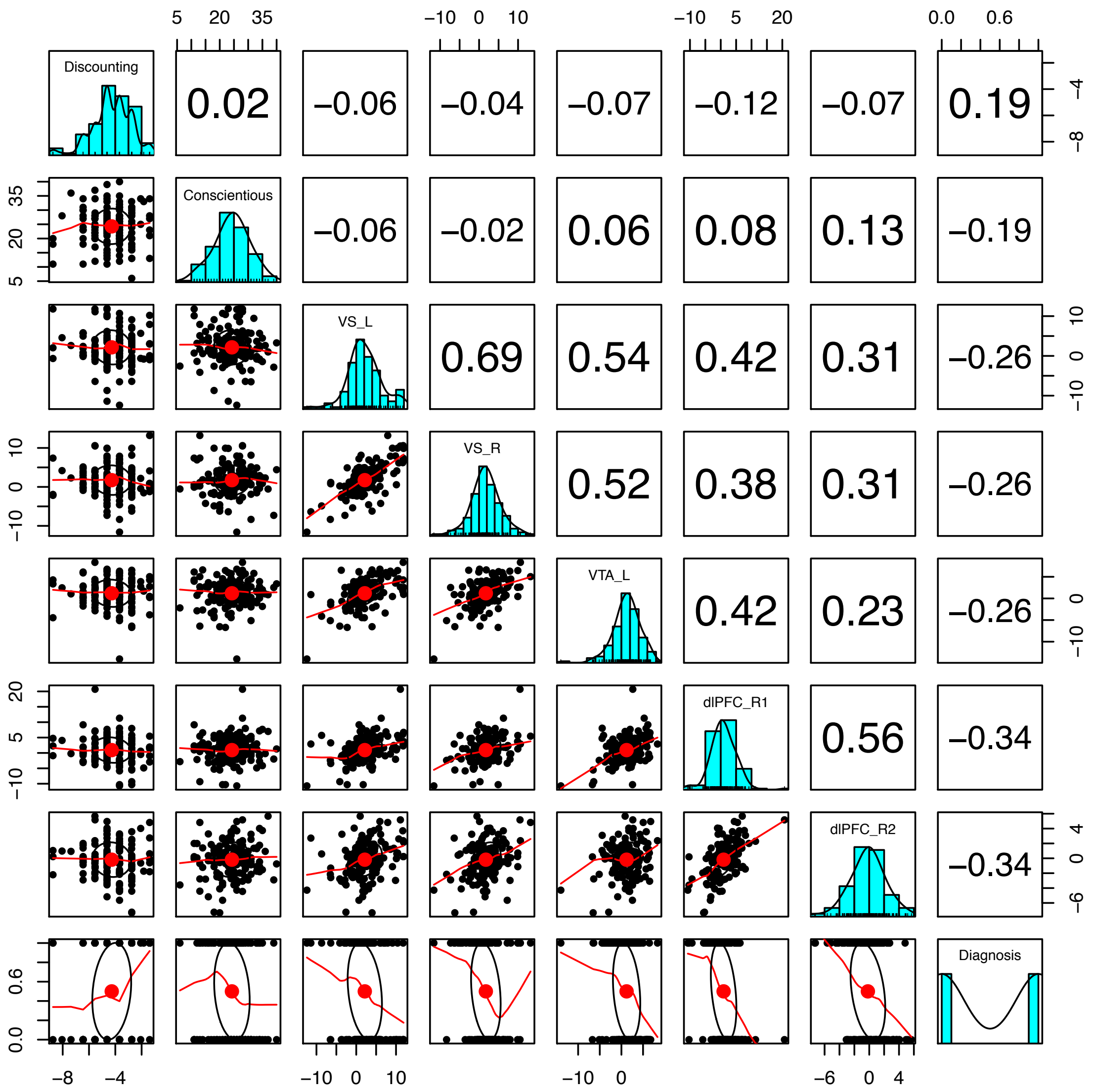

Supplementary Figure 7. Bivariate associations between independent variables and the dependent variable of diagnosis (VS_L: left ventral striatum; VS_R: right ventral striatum; VTA_L: left ventral tegmental area; dlPFC_R1: right dorsolateral prefrontal cortex 1; dlPFC_R2: right dorsolateral prefrontal cortex 2; Diagnosis: Control versus Problematic Drug Use Group).

	Psychological	Neural	Combined
Delay Discounting	0.28 (0.12)*<br>2.30		0.28 (0.13)**<br>2.19
Conscientiousness	-0.07 (0.03)*<br>-2.27		-0.06 (0.03)*<br>-2.00
L Midbrain		-0.16 (0.07)*<br>-2.44	-0.16 (0.07)*<br>-2.36
R Dorsolateral Prefrontal Cortex		-0.32 (0.09)**<br>-3.49	-0.33 (0.10)**<br>-3.36
Pseudo R^2 (ML)	0.07	0.15	0.20
AIC	195	181	176
Classification %	58 / 55	67 / 65	64 / 60

Statistics are standardized coefficients, followed by standard errors of the mean in parentheses, and Z-scores (n=144, significance: *$p < 0.05$; **$p < 0.01$, t-test). ML: Maximum Likelihood; AIC: Akaike Information criterion.

Classification was determined using 10-fold cross-validation over fits of a linear support vector machine, with 50% classification representing chance (train/test rates).

Supplementary Table 1. Logistic regression models of psychological and neural features predicting problematic drug use in novelty-seeking adolescents two years later.
